# Advanced Nanomaterials-Based Electrochemical Biosensors for Catecholamines Detection: Challenges and Trends

**DOI:** 10.3390/bios13020211

**Published:** 2023-01-31

**Authors:** Zina Fredj, Mohamad Sawan

**Affiliations:** CenBRAIN Neurotech, School of Engineering, Westlake University, Hangzhou 310030, China

**Keywords:** electrochemical biosensor, nanomaterials, catecholamine neurotransmitters, dopamine, epinephrine, norepinephrine

## Abstract

Catecholamines, including dopamine, epinephrine, and norepinephrine, are considered one of the most crucial subgroups of neurotransmitters in the central nervous system (CNS), in which they act at the brain’s highest levels of mental function and play key roles in neurological disorders. Accordingly, the analysis of such catecholamines in biological samples has shown a great interest in clinical and pharmaceutical importance toward the early diagnosis of neurological diseases such as Epilepsy, Parkinson, and Alzheimer diseases. As promising routes for the real-time monitoring of catecholamine neurotransmitters, optical and electrochemical biosensors have been widely adopted and perceived as a dramatically accelerating development in the last decade. Therefore, this review aims to provide a comprehensive overview on the recent advances and main challenges in catecholamines biosensors. Particular emphasis is given to electrochemical biosensors, reviewing their sensing mechanism and the unique characteristics brought by the emergence of nanotechnology. Based on specific biosensors’ performance metrics, multiple perspectives on the therapeutic use of nanomaterial for catecholamines analysis and future development trends are also summarized.

## 1. Introduction

Neurotransmission is critical for healthy brain functioning, memory, learning processes and even supports daily life [1,2,3]. In the process of neurotransmission (synaptic transmission), neurons use neurotransmitters (NTs) to afford communication between each other and their target tissues. While depending on their molecular structure, mechanism activity (direct or as a neuromodulator), and physiological function (excitatory or inhibitory), more than two hundred NTs have been identified to be implicated in synaptic transmission [4]. These small molecules and hormones act at the brain’s highest levels of mental function. Thus, abnormal levels of several NTs have been associated with numerous neurological and psychiatric diseases such as Parkinson’s [5], Schizophrenia [6], prion [7], and Alzheimer’s [8]. Whereas their autoxidation has been recognized as one of the potential trigger factors for dopaminergic neuron loss [9], making their role in specific mental disorders an active research topic during the last decades.

**Figure 1:**
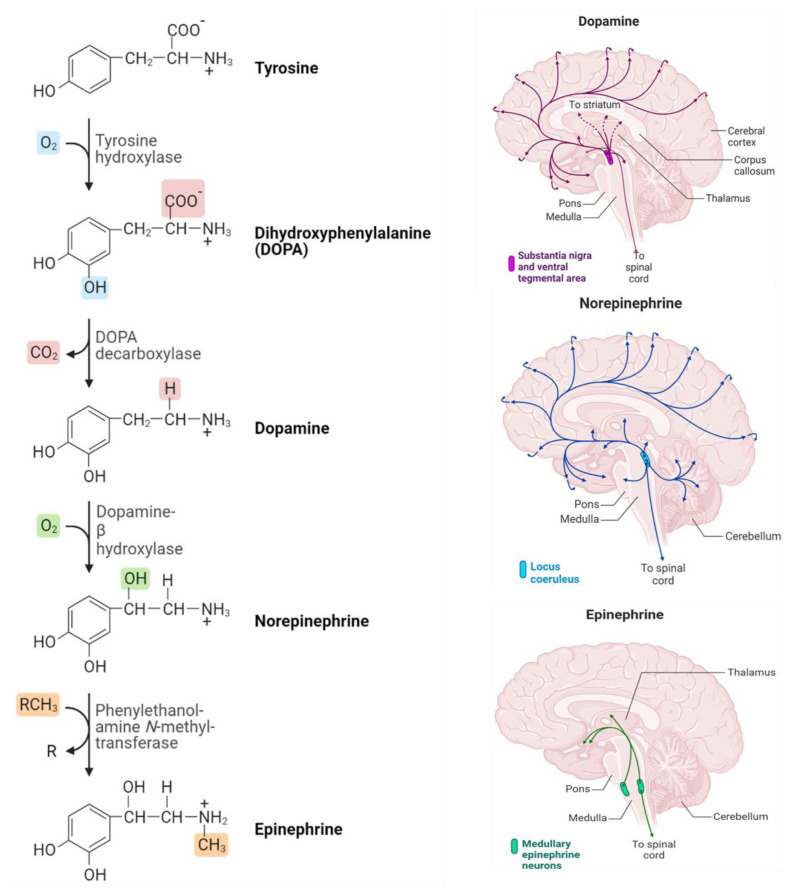
Biosynthetic pathway and distribution of catecholamine neurotransmitters in the human brain (Produced using BioRender).

Based on the chemical structure of these NTs, one specific type issued monoamine class also belongs to tryptamines (serotonin and melatonin), and histamine (phenethylamines) is called catecholamine. Dopamine, epinephrine (adrenaline), and norepinephrine (noradrenaline) whose chemical structure is summarized in Figure 1 and represents the three main NTs of catecholamines [10]. These neurotransmitters are composed of benzene rings, two hydroxyl groups, and an amine in their side chains. Catecholamine neurotransmitters perform a fundamental role in various processes, such as learning, emotion, memory, motor control, and regulation of the endocrine system. Similarly, noradrenaline and adrenaline play key roles in the stress response [11]. Additionally, current research revealed that catecholamines control significant functions in the physiological regulation of the immunological, respiratory, cardiovascular, and metabolic systems in the most severely affected COVID-19 patients [12]. Hence, minimizing the COVID-19 severity-related epinephrine effect was previously discussed [13].

Therefore, real-time monitoring of the catecholamine NTs in human body fluids, like serum, plasma, and saliva, will advance the research development in the field of neurological disease treatment, making it a promising avenue for early disease diagnosis. When it comes to neurotransmitters-based brain microdialysis measurement [14], high-pressure liquid chromatography [15], mass spectroscopy [16], and capillary electrophoresis [17] are mostly performed. While there is high sensitivity and specificity of these detection techniques, they still exhibit a heavy, expensive, and complicated setup to be generalized to the entire population. Therefore, a constant update of the current detection platform to meet the growing challenges of disease diagnosis, where the research and development of novel detection platforms with high sensitivity, low limit of detection, rapid, flexible, inexpensive, and easy to implement without any interference and toxicity of the sensing platform for early diagnosis is still an urgent need. To date, electrochemical analytical techniques, with their appealing limit of detection down to femtomolar, a fast response time of sub milliseconds, the ability to maintain cell viability during cell implantation, and the advantage of being a powerful tool for intracellular detection, have renewed increasing attention to biosensing design [18]. However, simultaneous detection of catecholamines is a particular challenge because of their similar molecular structures and physicochemical properties, making it difficult to differentiate between them. Given the significance of real-time NTs monitoring, to date, a couple of potential reviews have been reported. For instance, Lakard et al. highlighted the electrochemical biosensing approach of dopamine monitoring [19]. Another comprehensive summary of recent advances analytical tools based on optical and electrochemical techniques for dopamine detection was discussed and reviewed by Lakshmanakumar et al. [20]. In addition, update to in vivo real-time sensing techniques of neurotransmitters was recently reviewed by our group [21]. Other authors summarized technical challenges and obstacles in the development of NTs monitoring devices [22]. The reviews, which is limited to one catecholamine neurotransmitter (dopamine), updates to biosensors for epinephrine and norepinephrine and the associated challenges toward multi-detection were lacking. Moreover, emphasizing the advanced nanotechnology covering most trending electrochemical biosensors applications still needed to be offered.

Our goal in this review is to provide an overview of recent developments in electrodes modified with nanomaterials, which are efficient for the highly sensitive and selective electrochemical detection of catecholamines. Among the numerous available nanomaterials, we concentrated on the following four main groups: Carbon nanomaterials, particularly graphene oxide, and carbon, metal and metal oxide nanomaterials, and conductive polymers, all of which can enhance the electrocatalytic activity of catecholamines. Additionally, different types of catecholamine biosensors, including enzymes and aptamer-based bio-platforms, are described. In addition, some developed optical strategies are reviewed and compared with electrochemical biosensors.

## 2. Catecholamines Significance in Biological System

Commonly, the three NTs (Dopamine, Epinephrine, and Norepinephrine) are sequentially synthesized by enzymatic reactions in the biological system. Where the catecholamines synthesis begins with the amino acid tyrosine. The process of the DOPA decarboxylase converts DOPA into the amino acid dopamine, which is then converted into the hormone norepinephrine by the dopamine β-hydroxylase. Eventually, norepinephrine is converted into epinephrine through the action of the enzyme phenylethylethanolamine N-methyltransferase (PNMT). Figure 1 illustrates the synthesis process that takes place and how catecholamine neurotransmitters are distributed in the brain [23].

Dopamine, also known as 3-hydroxytryptamine, is accumulated within synapses and released by synaptic vesicles [24]. Carlsson and colleagues discovered its function as a neurotransmitter in 1958 at the Swedish National Cardiological Council Laboratory. This group received the Nobel prize in physiology and medicine since he has demonstrated that dopamine is not only a precursor of norepinephrine and epinephrine but also a neurotransmitter [25,26]. After the discovery of DA, it has been well studied how it works in the central nervous system, kidneys, cardiovascular system, and hormonal system [27,28,29]. It also supports emotional stability and hormonal balance, while low DA levels can lead to stress and depression [30]. It has also been found that dopamine is associated with the reward system, the brain’s circuit responsible for stimulus-seeking behavior, and emotions related to satisfaction and satiation. The clinical concentration of DA is generally very low, for instance, DA levels in urine, plasma, and a single adrenal chromaffin cell are in the order of μM, nM, and fM, respectively [31]. Norepinephrine (NE), also known as noradrenaline, is the primary neurotransmitter of the parasympathetic nervous system (PNS), influencing the immune system, most visceral organs, and glands [32].

NE is also an important CNS neurotransmitter. Previous research has reported that NE contributes to physiological and behavioral responses to stress [33].

Furthermore, it provides several effects on the body, the most important being associated with the “fight or flight” response to perceived danger [34]. A strong correlation between depression and NE concentration has been demonstrated in previous research. Depression has become the world’s leading cause of illness and results in high morbidity, mortality, and disability [35]. Epinephrine (EP) is a hormone and neurotransmitter in the mammalian central nervous system essential for the normal functioning of the CNS, renal, hormonal and cardiovascular systems [36]. The normal concentration of EP present in the plasma or blood fluids is approximately between 0.09 and 0.69 ng mL^−1^, and in some cases, during certain diseases, its level red a peak of 10 to 50 times higher than normal [37]. Any imbalance in EP concentrations has been linked to various diseases, including chronic active hepatitis, adrenal hyperplasia, and hypoglycemia. Additionally, stress and thyroid hormone deficiency are associated with high epinephrine levels [38]. In contrast, the decreases of EP concentration in the body are diagnostic criteria for mental disorders such as Parkinson’s [39], anxiety syndrome [40], panic attacks [41], and post-traumatic stress [42]. For all these reasons, the sensitive determination of epinephrine has received great attention in the last two decades.

## 3. Nanomaterials Enhancing Electrochemical Biosensors for Catecholamines Detection

**Figure 2:**
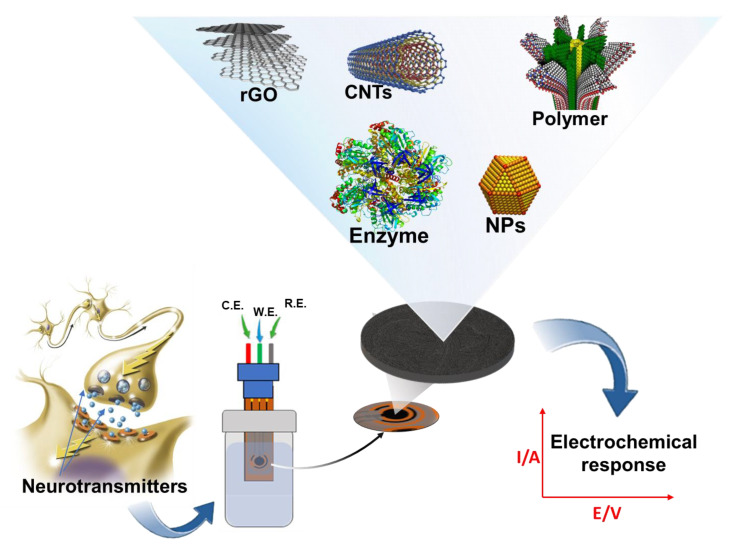
Simplified diagram showing electrochemical sensors/biosensors following various surface modifications for catecholamine detection.

In this section, we review the recent advances in electrochemical sensors for catecholamine sensing over the last five years, with a special emphasis on the highly innovative features introduced by advanced nanomaterials. We classify and summarize electrochemical sensors according to their substrate surface modifications. Further, we discuss electrochemical catecholamine sensors’ current and future states in terms of analytical device performance and emerging applications. Figure 2 shows a summarized schematic of this section of the proposed review. Over the last two decades, biomedical research has focused on the development of a miniaturized electrochemical platform to diagnose several brain diseases by rapidly detecting neurotransmitters and biomolecules related to the nervous system. The application of nanotechnology, based on advanced nanomaterials, in combination with electrochemical techniques, plays a key role for in vivo and in vitro monitoring of neurotransmitters in the earlier stages of brain diseases.

### 3.1. Carbon Nanomaterials Based Electrochemical Sensors

Based on carbonaceous nanomaterials’ attractive physicochemical and biological properties and their several derivatives, these nanomaterials have been used widely for neurotransmitter analysis [43]. Accordingly, they are suited for biosensor transducers to improve signal acquisition due to their unique properties, including their large surface-to-volume ratio, excellent chemical stability, high electrical conductivity, robust mechanical strength, ease of functionalization, biocompatibility, and biodegradability.

Due to these important properties, utilizing carbon-based nanomaterials as a catalysis probe has resulted in better sensitivity, wide linear detection range, low detection limit, and reusable sensors compared to conventional sensing techniques [44].

Recent studies have highlighted the unique physicochemical properties of carbon nanomaterials, including graphene, graphene oxide and carbon nanotubes (CNTs) for the development of a new generation of regulated, and improved electrochemical sensors for the detection of dopamine, epinephrine, and norepinephrine. A very low detection limit was achieved in the range of nanomolar in biological fluid, as reported in Table 1. These nanomaterials have been widely used as signal enhancements in different approaches to providing an effective platform for sensitivity improvement. One of the main achievements in developing selective electrochemical sensors has been using carbon nanotubes coated with a conductive polymer. Different strategies for ultrasensitive detection catecholamines established on carbon nanotubes coated with polymer matrix have been developed [45,46]. In this context, a multiwalled carbon nanotubes mixed with Nafion polymer composite modified carbon electrodes for selective DA detection in the presence of uric and ascorbic acid was demonstrated [47]. Indeed, members of Numan’s group have fabricated a nanocomposite based on multiwalled carbon nanotubes and cobalt oxide nanocubes as highly responsive platform for amperometric determination of DA with detection limit of about 0.176 nM [48]. A cost-effective point-of-care (POC) device based on three-dimensional graphene(3DG) and CNT modified screen-printed gold electrode surface was introduced as a novel electrochemical sensor. To monitor the response of the developed sensor, a portable potentiostat based on microcontroller was fabricated. The developed portable approach was able to detect ascorbic acid, dopamine, and uric acid with LOD about 2.5, 0.4, 0.6 µM respectively [49]. Next, an electrochemical sensor for epinephrine detection was developed using a modified glassy carbon electrode with mesoporous carbon and nickel oxide (OMC-NiO) [50]. Electrochemical techniques, such as impedance spectroscopy, cyclic voltammetry and differential pulse voltammetry were used to explore the electrochemical behavior of the chemically modified sensor. The resultant peak current of the developed structure of OMC-NiO/GCE was significantly higher than those of OMC/GCE and bare GCE, suggesting a better electrocatalytic and detection activity of OMC-NiO/GCE. Further, the Nyquist plot also confirmed the improvement of the catalytic properties of OMC after the NiO nanocrystals incorporation. Whereas the DPV was finally performed for the detection of EP at a linear range of concentration from 0.8 μM to 50 μM, leading to a LOD down to 85 pM and a LOQ 0.37 μM.

Besides the carbon nanotube, the discovery of graphene in 2004 has led to considerable interest in this nanomaterial for its wide range of applications in biotechnology, including the development of high-performance electrochemical devices. Both graphene and reduced graphene oxide have been widely used as probe materials for catecholamine monitoring. Some of the related publications are listed in Table 1. Due to its high surface area, biocompatibility, and abundance of oxygen-containing functions, graphene oxide is frequently used in various applications. Members of Kiranmai’s group have proven that incorporating inorganic particles and rGO sheets appears to have the potential to improve sensing performance. A simple electrospinning method followed by a hydrothermal technique was established to create a TiO_2_-rGO nanocomposite for the detection of epinephrine [51].

The constructed ultrasensitive sensor achieved a low detection limit of about 8.11 nM, and sensitivity calculated to 0.126 µA µM^−1^. Due to overlapping oxidation potentials and electrode contamination, simultaneous detection of EP and serotonin (5-HT) cannot be performed on a carbon paste electrode (CPE). To overcome these weaknesses, a layer of rGO was deposed to the surface of the electrode, and good separation was demonstrated [52]. The modified structure achieved a lower LOD of about 0.33 and 3.99 nM for EP and 5HT, respectively. Moreover, the rGO-CPE structure showed good sensitivity to real sample analysis, high repeatability, and great stability.

To increase the device sensitivity, Kshipra et al. [53] recommended the use of a glassy carbon electrode (GCE) modified graphene oxide sheets and chemically bonded melamine (MGO) for the quantification of epinephrine. It has been shown that the electron transfer sites of MGO can be predicted by functional density theory. The authors confirmed that the electron-rich π-electron cloud as well as their electron-deficient regions were useful in supporting the redox reaction during the electrochemical reduction and oxidation processes. Furthermore, the terminal amino group groups contributed to the anchoring of the EP on the electrode surface. After modification, the electrode demonstrated satisfactory sensitivity in the range of concentrations from 100 µM to 600 µM with an LOD of 0.13 μM. Suriyaprakash et al. [54] fabricated for the first time a POC flexible device for EP monitoring based on rGO. In their work, epinephrine was detected in real samples at 20 pM as a low detection limit with a fast readout (2.2 s). The developed biosensor provided a 60-days lifetime with 95% stability over 25 cycles. To this end, it was determined that several different carbon/organic nanocomposites have been investigated by exploiting their π-electron mobility to increase the sensitivity of the device. However, improved sensor stability is still needed. All the previously mentioned work has confirmed that the design of electrochemical sensors based on carbon nanomaterials can provide an improved ability to evaluate NTs, even at low concentrations with a fast response compared with traditional techniques. Multidetection platforms of similar NT structures require more complex immobilization chemistry, which necessitates the immobilization of multiple probes on a single electrode surface. In this context, Thondaiman et al. [55] have foxed on the development of the amperometric approach for the simultaneous quantification of DA and EP.

**Figure 3:**
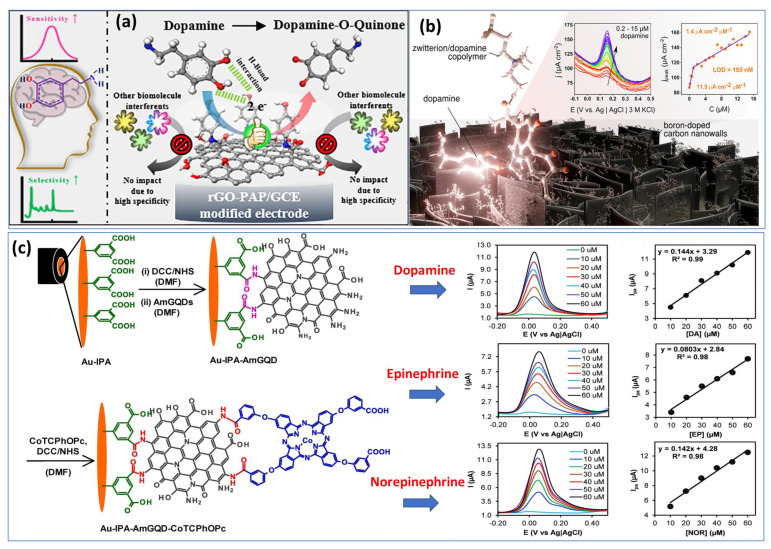
Examples of biosensors: (**a**) rGOPAP based sensor for the specific detection of DA. Reproduced with permission from [57]. Copyright 2021, ACS. (**b**) Electrochemical sensor based on copolymer electropolymerized at carbon nanowalls for sensitive recognition of DA. Reproduced with permission from [58]. Copyright 2021, ACS, and (**c**) preparation processes of the Au−IPA−AmGQD−CoTCPhOPc structure for catecholamine monitoring with varying concentrations from 10 µM to 60 µM for DA, EP, and NE in sera samples. Reproduced with permission from [72]. Copyright 2023, Elsevier.

Utilizing basic chemical oxidation and electrodeposition methods, a surface-modified copper mesh electrode was created using a heteroatom-doped GQD-assisted conductive polymer (PEDOT). The so-called multidetection system has been performed with two separate working electrodes. Additionally, in this work, the LOD was calculated to be approximately 0.27 μM and 0.084 μM for DA and EP, respectively, which are considered high compared with individual detection. Subsequently, another research group has overcome the previous drawback by proposing a two-in-one approach for detecting epinephrine and norepinephrine using an electrochemically activated pencil graphite electrode. A mixture of analytes showed that EP and NE peak currents increased with concentrations between 2.5 μM and 250 μM. In human plasma samples, levels of 0.83 μM were determined for NE and 0.99 μM for EP [56]. Most work published so far has focused on the individual detection of a single catecholamine neurotransmitter species. We took the example of two different approaches proposed recently for the detection of DA based on reduced graphene oxide and p-aminophenol (Figure 3a) [57] and boron-doped nanowalls with an electropolymerized polydopamine/polyzwitterion (Figure 3b) [58].

**Table 1:** Comparison of electrochemical catecholamines sensor-based carbon nanomaterials and its derivatives.

NTs	Sample	Catalyst/Transducer	Technique Used	Linear Range (μM)	Detection Limit (nM)	Ref.
DA	Human serum	MWCNTs-ZnO/GCE	CV, DPV	0.01–30	3.2	[59]
DA, 5-HT	PBS	Curcumin oxidized carbon nanotubes/GCE	LSV	0–170 10–130	0.010 0.011	[60]
EP	Real water	MWCNTs-molybdenum disulphide/GCE	CV	9.9–137.9	0.003	[61]
DA, EP	Synthetic urine	Oxidized capsaicin-MWCNTs/GCE	CV Amperometry	5–75 5–115	0.0072 0.0015	[62]
EP	Urine and pharmaceutical sample	Chitosan-functionalized carbon nanotubes/GCE	CV, DPV	0.05–10	30	[63]
DA	Human blood serum	CaCO_3_-PANi-rGO/GCE	DPV	0.1–14	100	[64]
DA, UA	PBS buffer	Thermally rGO/GCE	CV, DPV	5–42	120 150	[65]
DA	Human urine	rGO-tungsten trioxide/ GCE	CV, Amperometry	0.3–1245	306	[66]
EP	PBS buffer	rGO-MoS_2_-Fe_3_O_4_/GCE	CV, DPV	0–11	137	[67]
EP	Human serum	2D nickel oxide-rGO/GCE	CV, DPV	50–500	1000	[68]
EP	Urine	rGO-Ti_3_C_2_Tx MXene/Indium tin oxide	DPV	1–60	3.5	[69]
DA	PBS	GO-CuAlO_2_/GCE	LSV	0.92–10	15	[70]
EP	Human serum	Au-Pd-rGO/GCE	CV, DPV	0.001–1000	12	[71]

However, few groups have succeeded in the fabrication of sensors for the detection of the three catecholamine NTs (DA, EP and NE) caused to their similar chemical structures. Recently, members of the Luhana’s group [72] have developed an approach based on a covalent conjugate aminated graphene quantum dots (IPA−AmGQD) and carboxylic acid cobalt phthalocyanines (CoTCPhOPc) attached onto gold electrode (Figure 3c). From the cyclic voltammetry diagrams of the three catecholamines, almost similar oxidation and reduction potentials are observed, with a slight shift. In terms of intensity, dopamine shows the largest current peak, most likely due to its greater adsorption at the surface of electrode/electrolyte. Finally, carbonous nanomaterials have shown a good capability for generating very different structures with properties allowing for individual and multi-detection of catecholamines in a variety of biological environments. Besides, the integration of carbon nanostructures into other nonmaterials such as organic polymers or hydrogels or coupling them to metal or oxide nanoparticles is a promising approach to increase the sensing ability of sensors or to improve their performance.

**Figure 4:**
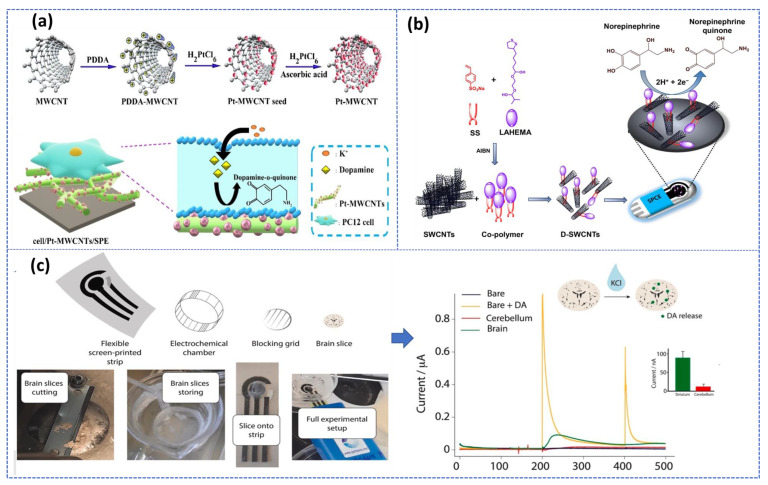
Ex vivo electrochemical biosensors based SPE: (**a**) Monitoring of DA secreted from PC 12 cells using Pt nanoparticle decorated CNTs. Reproduced with permission from [73]. Copyright 2023, Elsevier, (**b**) Schematic illustration of NE quantification in rat tissue samples based on Co−Polymer and SWCNTs. Reproduced with permission from [74]. Copyright 2023. Elsevier, and (**c**) experimental setup of DA detection released in mice brain using unmodified SPE and its corresponding chronoamperometric measurements with different configurations for striatum cerebellum brain slices before and after 0.1 M KCl stimulation. Reproduced with permission from [75].

In addition, carbon nanostructures can be integrated into nano/micro-scale devices, such as microfluidic chips or microelectronic devices, which are particularly promising for the fabrication of multiple analyte sensing devices and miniaturized sensors. Later, these advances in biosensor research open interesting prospects for the development of promising platforms for real-time monitoring of multi-neurotransmitters in biological samples. More critically, this electrochemical sensor-based carbon nanomaterial was performed to monitor in real-time the DA released by living PC12 cells with very high efficiency [73]. Also, carbon nanotube coated with conducting polymers based integrated nanobiosensors was used for detecting epinephrine in ex vivo rat tissue [74]. Additionally, Verde et al. [75] reported an Organ-on-screen-printed approach to monitor dopamine release in mice’s brain based on a flexible screen-printed platform (Figure 4c). After optimization of the experimental condition in both in buffer and PC-12 cell culture media, the printed strip provided an LOD of about 1 μM and a linear range up to 160 μM.

As demonstrated, the three-approach presented in Figure 4 recommended the use of the screen-printed electrode (SPE), while SPEs are disposable, inexpensive, and reproducible devices that can be easily manufactured in bulk without any preprocessing steps, allowing for real-time, in-situ detection. Therefore, these three electrodes printed on a single chip are considered promising alternatives to develop a non-invasive, portable, cost-effective detection system with high sensitivity and specificity required for POC diagnosis. In this context, multiple reviews have been published recently summarizing the benefits of using SPEs for engineering electrochemical biosensors [76,77,78].

Recently, a specific subclass of SPE called laser-induced graphene (LIG) electrode through polyimide sheets (PI) conversation to graphene using directed induced laser is becoming increasingly popular. LIG method is known for being mask-free and easy to use, offering a cost-effective and efficient means of producing diagnostic kits for on-site testing compared to most graphene manufacturing techniques [79]. LIG structure is typically created following a photothermal pyrolysis process. Consequently, the obtained structure is usually displaying defects with intricate porosity patterns. These defects were found to be beneficial for immobilizing probes and increasing sensitivity [80]. Accordingly, Xu et al. [81] used the laser-scribed graphene grass structure, manufactured onto a simple plastic-polyimide (PI) film via laser irradiation for the simultaneous detection of DA, EP, and NE within a low LOD of 0.43 µΜ, 1.1 µM, and 1.3 µM, respectively. Interestingly, the suggested disposable biosensor still maintains high sensitivity and selectivity in human serum and in the presence of UA and AA as potential interferants. However, a lower LOD of 7 nM was reported by Berni et al. [82], in which they demonstrated the DA monitoring by coupling the unique properties of LGE with the excellent cation exchange characteristics of the polypyrrole film. The detection strategy provides outstanding selectivity and sensitivity, even in the presence of a high excess of AA.

### 3.2. Metal Nanoparticles-Based Sensors

To date, noble metals such as gold, silver, copper, and metal oxide NPs have attracted considerable attention in electrochemistry due to their ability to detect and amplify various signals. Thereby, several reported works have recommended the immobilization of AuNPs on the electrode’s surfaces to enhance the electrical signals of catecholamines electrochemical detection because of their simple preparation, high surface-to-volume ratio, electrocatalytic ability, and chemical stability [83,84]. The electrocatalytic redox activity of dopamine was improved using gold nanoparticles systematically decorated with Fe_3_O_4_ magnetic nanocomposites to obtain detection limit of 2.7 nM [85]. Later Lim’s research group confirmed mixing AuNPs, and the multilayer of carbon nanomaterials (CNT) provided excellent biostability and high-performance electrochemical sensing capability [86].

Recently, Zhan et al. [87] used gold nanoparticles and polydopamine to modify a free-standing acupuncture needle microelectrode for detecting EP. The authors suggested using the acupuncture needle microelectrode, which is receiving increasing interest because of its convenience, easy fabrication process, reasonable price, high conductivity, and small volume of analyte during measurement. The developed probe has been successfully used to analyze EP in real human serum and provide real-time detection of adrenaline secreted by PC12 cells. Table 2 summarizes the main recent works in which metal and metal oxide nanoparticles modified electrodes surface have been applied to quantifying of catecholamines neurotransmitters. Since the limit of detection is very important due to the relatively low concentration in the patient sample, more attention was focused on testing sensitivity within a lower concentration range.

Additionally, typical two-dimensional materials, such as molybdenum disulfide (MoS_2_), have received much attention based on their unique structure as well it physical, chemical, and electronic properties [88]. According to Wu et al. [89], a nanocomposite that incorporates manganese ferrite nanoparticles and MoS_2_ could be used for the electrochemical multi-detection of three NTs.

Experimental studies using DPV technique have shown that the developed nanocomposite can detect AA, DA, and UA individually, simultaneously, and in a relatively wide linear range.

**Table 2:** Comparison of electrochemical catecholamines sensor based on metal and metal oxide NPs.

NTs	Transducer	Probe	Detection Method	Linear Range (μM)	LOD (μM)	Ref.
DA	PGE	Citrate-stabilized gold nanoparticles @polydopamine)	SWV	0.5–7.0	0.53	[90]
DA	GCE	Copper nanoparticles	CV, DPV	0.05–5.0	0.04	[91]
DA	GCE	Carbon quantum dots and copper oxide	SWV	1–180	25.4	[92]
DA	Diamond anoporous	AuNPs and Nafion	SWV	3–100	0.068	[93]
DA	GCE	Gold-decorated porous silicon-poly(3-hexylthiophene)	Amperometry	1–460	0.63	[94]
DA, UA	CPE	Cu-based metal-organic frameworks	DVP	0.05–500	0.03 0.07	[95]
DA	GCE	Copper organic framework@halloysite nanotubes-rGO	DPV	0.1–130	0.015	[96]
DA	GCE	Carbon-titanium nitride nanoparticles	DPV	0.1–250	0.03	[97]
DA	GCE	Palladium nanoparticles decorated nickel-based metal–organic framework	CV, DPV	0.001–100	0.01	[98]
DA	GCE	Nitrogen-doped titanium dioxide-AgNPs-GQD	CV, DPV	0.003–300	0.001	[99]
DA	FTO	Nanoplatelets of zinc oxide embedded polyvinyl alcohol	EIS	0.020–3000	0.005	[100]
DA	GCE	Cobalt phthalocyanine-nitrogen-doped GQD	Amperometry	100–1000	0.12	[101]
DA	Carbon spheres	Sodium tungsten bronzes nanoparticles	DPV	0.004–106.4	0.001	[102]
EP, NE	CPE	Cu quantum dot@ SH-nanoparticles immobilized on CuMOF	DPV	0.2–34,285	1.6 0.5	[103]
NE	GCE	Graphene quantum dots decorated AuNPs	DPV	0.5–7.5	0.15	[104]
DA, EP	CPE	Nickel telluride	SWV	4–31	0.15 0.35	[105]

#### MOF and COF-Based Sensor

Metal-organic frameworks (MOFs) are considered a subset of coordination polymers made of metal ions or clusters and organic ligands. A variety of techniques were adapted for MOFs preparation, including hydro-/solvothermal methods, mechanochemical, microwave-assisted, sonochemical, and electrochemical [106]. Such nanocomposites feature the potential of adjustable porosity structure that hangs on the synthetic conditions, organic and metal sources, and post-synthetic modification; hence, a great diversity of MOFs might be issued [107]. Not to mention their outstanding ordered structures, high surface area, and exceptionally adaptable functionalities that suit various fields, such as contaminant sorption, gas storage and separation, drug delivery, environment, and biological sensing [108,109]. To date, there has been an increasing interest in MOFs as promising electrode materials for electrochemical sensing applications due to their porous structure that increases the surface area, hence improving the transport and adsorption of the analyte. Additionally, the variable-sized and shaped pores within the MOFs lead to better selectivity for certain analytes. Lastly, the abundance of functional sites, such as metal centers, linkers, and active guests, enhance the analyte adsorption, activation, and direct electron transfer. When it comes to the electrochemical quantification of NTS, MOFs supported with conductive nanomaterials have been widely performed [110]. Giving the example of Liu’s group [111] work, in which authors prepared for the first time the MOF-235 using a single-step hydrothermal method for the simultaneous detection of dopamine and uric acid within a low LOD of 3.34 μM and 3.46 μM, respectively. To fabricate the sensing platform, the synthesized MOF was drop-cast on the surface of a glassy carbon electrode. Further, the MOF-235 was endowed with distinct functional groups resulting in efficient heterogeneous electron transfer, which facilitates the highly selective determination of analytes with well-separation packs. Such a hierarchical 3D structure (MOF-235) was found to be advantageous in enlarging the surface area, thus uplifting its potential application in the electrochemical monitoring of small molecules related to the medical field. Newly, Fallah et al. [103] have benefited from the advantageous characteristics of the metal-organic framework. They recently reported an electrochemical sensor for the detection of multi-neurotransmitters (NE, EP, and piroxicam) based on CeMOFs functionalized with Cu quantum dots (Cu QD) and SH-SiO2 nanoparticles. This new method of sensing was successfully applied to the detection of catecholamines in urine and plasma as biological samples with satisfactory results and a detection limit of less than 0.05 µM of piroxicam. Additionally, to confirm its reliability, the results obtained by the proposed sensor were validated using conventional high-performance liquid chromatography showing a good concordance between both techniques.

While the outstanding performances compared with other complicated methods, where several steps for surface modification still need to be performed, using MOFs have certainly expanded the electrochemical applications based on nanocomposites by providing a new sensing direction. However, covalent organic frameworks (COFs) have drawn much attraction because of their structural diversity and versatility, and unique stability. Combining the COF nanostructure and MWCNTs as a sensing interface, Guo et al. [112] suggested the development of a new detection platform for the simultaneous detection of dopamine and uric acid. The electrochemical sensor exhibits a strong current response to both DA and UA in phosphate buffer. Such a high sensing efficiency was referred to the synergistic effects of COFs and MWCNTs. Hence, the developed sensor could detect DA and UA in a wide linear range between 0.6 and 250 μM with low detection limits of 73 nM and 63 nM, respectively. Whereas Wang et al. [113] reported the usage of magnetic COF nanosphere [Fe3O4@COF@2-FPBA] based boronate affinity adsorbents for the detection of DA down to 0.31 ng mL−1 within a linear detection ranged between 2 to 200 ng mL−1. Additionally, using [Fe3O4@COF@2-FPBA] nanoparticles as adsorbents at neutral pH, the authors successfully extracted five monoamine neurotransmitters. While using these nanoparticles, a new analytical method was also reported through the combination of fluorescence detection with HPLC (HPLC-FLD) for monoamine neurotransmitters detection in urine.

### 3.3. Polymer Film Based Electrochemical Sensors

The physico-chemical properties of polymers have attracted considerable interest in the current design of electrochemical biosensors, especially for neurotransmitters monitoring [114]. Since the use of polymer structure for sensors development improved its performances by increasing the conductivity and the specific surface of the electrode. Table 3 shows some of the recent work in which conductive and non-conductive polymer modified electrodes surface have been applied to the quantification of catecholamines neurotransmitters. The most used procedure for the deposition of polymer films is the electropolymerization under galvanostatic, potentiostatic, or, more commonly, potentiodynamic conditions. The advantage of this technique is to create uniform films with precise thicknesses controllable onto the electrode surfaces. Furthermore, the high level of physical and chemical stability provided by the strong adhesion between the films and the active surfaces of the electrodes makes polymers a very efficient support for the immobilization of biomaterials [115,116].

Based on all the aforementioned features, Banu et al. [117] modified carbon paste electrode surface with a polymerized film of Victoria blue B monomer for the multi detection of epinephrine and serotonin in the presence of guanine and adenine using differential pulse voltammetry. The peaks are well separated, making it possible to analyze complex samples simultaneously with both analytes. The utilization of electropolymerised film for the analysis of catecholamines is based on the diffusion of the analyte at the electrode surface and several studies have demonstrated the ability of polymeric organic acid films for the determination of catecholamine NTs.

Fatma et al. demonstrated a new approach for the multi-detection of epinephrine and dopamine using one nonomer acryloylated graphene oxide-carbon black nanocomposite polymer [118]. To determinate the sensor’s performance, the voltametric responses were tested in buffer solution, urine, pharmaceutical samples and human blood. Under optimized experimental conditions, the developed sensor exhibits high sensitivity with detection limit of about 0.028 ng mL^−1^ for DA and 0.018 ng mL^−1^ for EP. It is important to note that a great number of molecularly imprinted polymer materials (MIPs) are used for electrode modification based the catecholamines electrochemical biosensor [119].

**Table 3:** Comparison of electrochemical catecholamines sensor using electrodes modified with polymers.

Catecho-Lamine	Transducer	Catalyst	Technique Used	Linear Range (μM)	Detection Limit (nM)	Ref.
DA	GCE	Poly paraphenylene diamine	DPV	0.038–4.76	0.094	[120]
DA, UA	GCE	polypyrrole matrix supported iron	CV	10–900	321 348	[121]
DA	GCE	polyaniline-WO_3_	CV, DPV	20–300	139	[122]
DA	CPE	Polymelamine-AuNPs	CV, DPV	0.2–11	67	[123]
DA	LSGE	Overoxidized polypyrrole (PPy_ox_)	CV, DPV	0.010–10	7	[82]
EP, 5-HT	CPE	Poly Victoria blue B	DPV	1–80	330 980	[117]
DA	GCE	Poly-tryptophan	DPV	0.2–100	60	[124]
DA, UA, AA	GCE	Copper monoamino-phthalocyanine-acrylate polymer	DPV	0.01–10	0.7 2.5 5	[125]

However, MIPs enabled the development of a novel flexible biosensor platform that has potential for application in the pharmaceutical field due to its simplicity, low cost, and portability [126]. They are synthesized directly onto the working electrode or attached to a solid support before being cast onto the electrode. Given the example of Zhang et al. [127], a nanocomposite based on molecularly imprinted polymers synthesis using poly(3-aminophenylboronic acid coted via electrochemical polymerization on MWCNTs was fabricated for the detection of epinephrine. Additionally, a poly(9-carbazoleacetic acid) based MIP was prepared for detecting EP and DA simultaneously in plasma samples [128]. Similarly, certain materials have been successfully used as supporting materials or as components of composites containing MIPs for the fabrication of electrochemical sensors for catecholamines. Among them, quantum dots can improve electrode sensitivity through their conductivity, while MIPs enhance electrode selectivity. When QDs were incorporated into an electrode modified with MIPs, very good sensitivity and detection limits were achieved [129,130].

Modification of electrodes with several advanced nanomaterials groups discussed above, including carbon nanotubes, metal oxide nanoparticles, graphene oxide, and polymer, has been used to detect catecholamines in the presence of various chemicals. Fortunately, these improvements have enabled the development of platforms capable of identifying catecholamines even in the presence of significant interferences such as AA and UA. By separating the potentials for detecting the analyte and the interferent, it is possible to simultaneously detect interferents and the analyte in some cases. In addition, the current response of the modified electrodes without and with interferents was measured to examine their resistance to interferent effects. When such interferents are present, the change in current response is commonly negligible.

## 4. DNA Aptamer-Based Catecholamine Biosensors

Bioreceptor selectivity is essential for the quantification of neurotransmitters in the complex brain environment. Alternatively, we found that discriminating catecholamine neurotransmitters is challenging because the molecules have overlapping chemical functions [131]. Electrodes functionalized with the aforementioned nanomaterials can provide enhanced surface area, while an affinity ligand is needed to increase selectivity. For such purposes, electrochemical biosensors based on aptamers have been the most successful strategy. Additionally, aptamers as recognition elements have renewed increasing attention in biosensing design owing to their cost effectiveness, small size, and chemical stability [132]. Whereas aptamer technology can overcome problems of poor selectivity due to its excellent ability to identify and bind to target molecules, this technology should also eliminate the interference from other molecules. More importantly, aptamers exhibit remarkable flexibility in their structure design, leading to novel biosensing platforms demonstrating high selectivity and sensitivity. Thus far, many approaches based on aptamers that recognize catecholamine neurotransmitters have been reported (Table 4).

**Figure 5:**
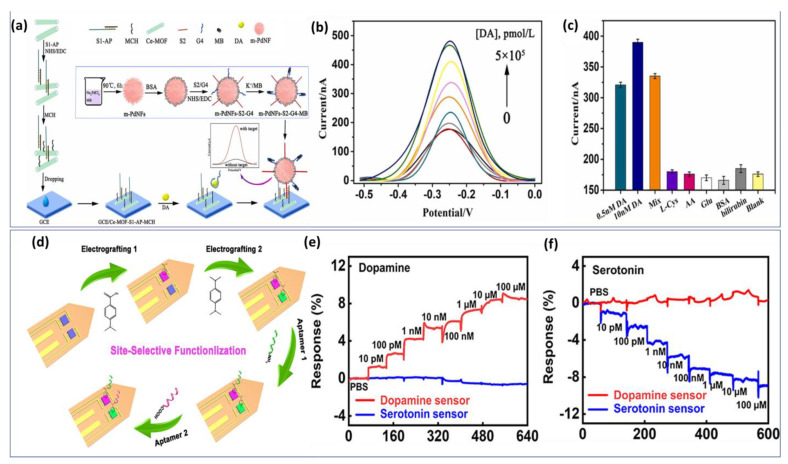
Assembly process of electrochemical aptasensor fabrication: (**a**) Methylene blue−integrated Ce−MOF capturing DNA for specific DA monitoring in human serum. (**b**) SWV curves of different concentrations of dopamine up to 100 nM. (**c**) Specificity study of the proposed aptasensor. Reproduced with permission from [133]. Copyright 2023, Elsevier, (**d**) Electrografting process of DNA−Aptamer-based multi-probe for simultaneous detection of DA and Serotonin in ex vivo. (**e**) Real-time response of dopamine and (**f**) serotonin with a concentration range from 10 pM to 100 μM. Reproduced with permission from [134]. Copyright 2022, ACS.

We have considered some representative work with potential application. Zhang et al. improved the performance of the dopamine aptasensor based on cerium metal framework (Ce-MOF) modified GCE [133]. Figure 5a demonstrates the fabrication process of the biosensor. First, a single-stranded nucleic acid (S1) with -NH_2_ terminal was arranged to cross interact with aptamer to form a double strand. Then, S1-Aptamer was linked to a Ce-MOF modified electrode. A second sequence S2 is attached to the signal material by a -CO-NH- linkage to form a signal probe. Using SWV curves (Figure 5b), the analytical performance of the aptasensor was evaluated for various concentrations of DA. The biosensing structure demonstrated a high affinity to DA detection with an LOD of about 6 pmol/L. In addition, several non-target interferents were selected to examine the analytical selectivity. Despite high concentrations of potential interferents, no significant current signal is detected (Figure 5c). Thus, the proposed biosensor exhibits a high level of specificity, providing a reliable experimental basis for the further analysis of clinical samples.

However, the drawback of this approach is the complex architecture of the detection platform requiring several modification phases. Most electrochemical biosensors based on aptamers have only been evaluated in buffer solution, and their performance in realistic biological environments remains unexplored. Such limitations represent a crucial challenge in their application in vivo. Therefore, these platforms are currently most suitable for studies in ex vivo, where biological samples can be treated to ensure optimal sensor performance. In addition, potential unspecific interactions between target molecules and AuNP surfaces are often not considered, which can lead to misinterpreted analyses. This issue was examined by Wu et al., where they demonstrate the capability of the implantable aptamer-graphene field-effect transistor (G-FET) probe for real time monitoring of dopamine release in vivo in mice models [135].

The developed biosensor exhibited a high selectivity, and picomolar sensitivity, simultaneously. Later, the same strategy based on soft neural probe was applied to the detection of multi-neurotransmitters via the electrografting-enabled site-selective functionalization of aptamers on G-FETs. The electrografted of tow functional groups −COOH and −NH_2_ serve as linkers to functionalize two aptamers with different functional groups (−NH2 and −COOH). Once the dopamine or serotonin binds to its specific aptamer, a conformational change in the functionalized aptamers on the graphene occurs, which alters the doping state of the graphene and results in a current changing on the source-drain of the GFET probe. Figure 5d illustrates the functionalization step by electrografting of G-FETs for multiplexed neurochemical monitoring in a single neuronal probe.

The multiplexed neural probes showed a high sensitivity for both dopamine (Figure 5e) and serotonin (Figure 5f) in ex vivo studies using harvested mouse brain tissue.

Despite the lower detection limit and high selectivity, aptamer-based biosensors technology still needs to be improved in terms of the modification process due to its complex chemistry. Indeed, the majority of aptamer-based biosensor was tested in ex vivo application. Therefore, they have great potential to be used in in vivo applications.

**Table 4:** Recently developed biosensors-based DNA aptamer technologies for catecholamine detection.

NTs	Biosensor Structure	Interferents	Sample	Measurement	Linear Range (nM)	LOD (nM)	Ref.
EP	Aptamer based Organic electrochemical transistors	DA, Cysteine, AA and tryptophan	PBS solution	Amperometry	0.9–90 × 10^3^	0.9	[136]
DA	Aptamer-AuNPs-rGO/GCE	AA, UA, EP and cathechol	Human serum	DPV	1–100	47	[137]
DA	Aptamer-Copper aluminate-rGO-TEPA/SPE	UA, AA, and glucose	Human serum	DPV	0.05–10 × 10^3^	0.017	[138]
DA	Aptamer-CeMOF/GCE	AA, BSA, and bilirubin	Clinical serum	SWV	0.5–100	0.06	[133]
DA	Aptamer-GCSC-GO/GCE	DOPA, AA, HVA	Human serum	DPV	1–1000	0.75	[139]
DA	Aptmer-Gold nanostructure/Au electrode	AA, UA, Catechol, EP, and NE	Clinical serum	DPV	0.163–20	0.01	[140]
EP	Aptamer-Methylene blue/GCE	AA, UA, and levodopa	SH-SY5Y cells	CV, DPV	200–10 × 10^3^	67	[141]

## 5. Enzyme Based Catecholamine Biosensors

Enzymes are often used as a recognition element for monitoring small molecule metabolites, as they have high catalytic efficiency and specificity and can rapidly convert substrates into products. For the quantification of catecholamines, enzyme-based biosensors have demonstrated good performance. The tyrosinase enzyme is the most popular enzyme that can function using an electrochemical or optical signaling mechanism. In this context, Sethuraman et al. [142] incorporated AuNPs and poly (thiophene-3-boronic acid) with tyrosinase enzyme (PPO) to monitor dopamine. Differential Pulse Voltammetry (DPV) technique determined the biosensor’s performances. Wide linear range of detection from 50 nM to 30 µM with an LOD of about 20 nM was obtained.

**Figure 6:**
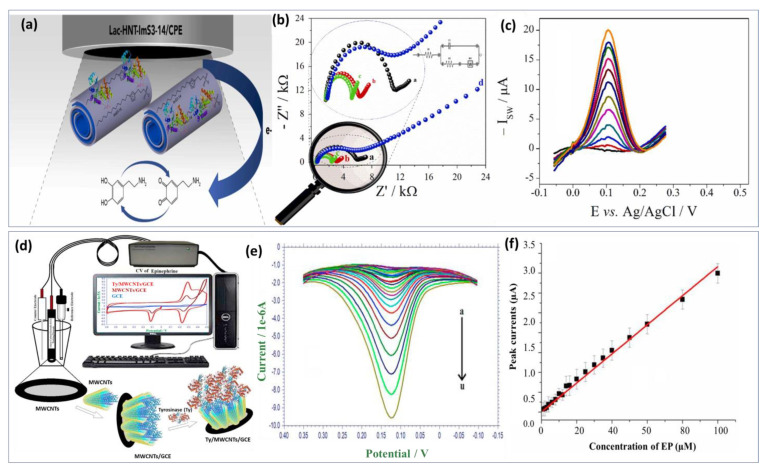
Enzyme based biosensor examples: (**a**) Schematic illustration of the biosensor based on laccase−halloysite nanotubes combined with imidazolium zwitterionic surfactants for dopamine detection. (**b**) EIS spectra in 5 mM [Fe(CN)_6_]^3−/4−^ prepared in KCl for different functionalization steps. (**c**) SWV curves obtained after incubation of different DA concentrations. Reproduced with permission from [146]. Copyright 2023, Elsevier. (**d**) Biosensing platform for epinephrine monitoring based on multi walled carbon nano tubes mediated tyrosinase enzyme. (**e**) DPV curves obtained at Ty/MWCNTs/GCE for different EP concentrations ranging from 3 μM to 200 μM. (**f**) Calibration curve of the obtained biosensor showing good sensitivity and reproducibility. Reproduced with permission from [147]. Copyright 2023, Elsevier.

Lately, tyrosinase enzyme have been immobilized on the AuNPs and La_2_O_3_ nanostructured modified indium-tin-oxide electrode surface [143]. Compared with the previous approach, long-term stability and reproducibility were achieved as well as fast response time under 30 s. Unlikely, the calculated detection limit was in the micromolar level (0.258 μM). Similarly, an innovative approach fabricated by Wu et al. [144] benefited from the advantage of the biocatalytic activity of laccase enzyme and the excellent conductivity of carbon quantum dots to develop a promising biosensor for DA detection. The detection mechanism involved dopamine adsorption on carbon dots via electrostatic interaction with the amine functional group. The developed enzymatic biosensor exhibits a low detection limit of about 80 nM and a wide linear range from 0.25 μM to 76.81 μM. Xie’s group [145] investigated the catalytic activity of a single-atom ruthenium-based biomimetic enzyme for the multi detection of DA and AA. In real biological serum samples, DA and UA were detected using the enzyme-based biomimetic biosensor with comparatively low detection limits of 20 and 170 nM, respectively, demonstrating good reproducibility and stability. However, enzyme biosensors face several challenges during the development process. Hence, screening out highly active enzymes and ensuring that the sensors have sufficient sensitivity is difficult. To achieve this, it is essential to ensure that the active enzymes are firmly anchored on the semiconductor chip. Additionally, the base film should be as thin as possible to reduce the response time and extend the sensor’s lifetime. Furthermore, the biosensor-based enzyme approach needs to be improved in terms of adaptability and stability. Recently, zwitterionic surfactants incorporated laccase enzyme was immobilized through physical adsorption on the surface of hylloysite nanotubes (HNTs) as shown in Figure 6a [146]. The obtained recognition probe modified carbon paste electrode provides enhanced catalytic activities and stability for dopamine biosensor (Figure 6b). It was known that HNTs are tubular nanomaterials with a large specific surface area and a high level of biocompatibility. Nonetheless, its use as a scaffolding to obtain a sensitive film with a higher specific surface area and more adsorption sites were well investigated. Hence, the combination of laccase and halloysite nanotube is a perfect approach for high sensitivity. Using this structure, a linear calibration plot between the DA concentrations and the peaks current was obtained (Figure 6c) with LOD calculated at 0.252 μM. Based on these achieved results, the proposed biosensor can be used in clinical applications as an effective new approach. Thereafter, a biosensor based on the physical adsorption of MWCNTs on the surface of the electrode followed by immobilization of tyrosinase enzyme was also reported (Figure 6d) [147]. Within this framework, authors focused on the study of the electrochemical redox mechanism of epinephrine. During the reduction process, two reduction peaks were noted at 0.181 V and −0.229 V. The effect of concentration on the voltammetric behavior of EP has been studied. Figure 6e illustrates EP’s DPV at different concentrations, with reduction currents increasing with increasing EP concentration. Based on Figure 6f, a good linear relationship was found between peak currents and EP concentrations. Besides the good stability and selectivity of the proposed approach, acceptable LOD was estimated to be 0.51 μM. In general, the immobilization process can directly impact all the enzyme biosensor performances like sensitivity, selectivity, and stability. Enzyme biomodification methods can be classified as follows: physical adsorption, covalent bonding, incorporation into a polymer matrix, and cross-linking [148].

In addition, the biocatalytic activity of enzymes depends critically on the sample solution’s applied potential, temperature, and pH. At the optimal condition, (pH, temperature, etc.) the highest reproducibility and sensitivity can be achieved [149]. Despite all the successful achievements and significant breakthroughs in the development and improvement of enzyme-based biosensors, there are still plenty of serious obstacles that need to be overcome. For example, the use of these biosensors in real-world monitoring applications is limited by their lack of high sensitivity, low stability and/or selectivity. For the rapid and automatic examination of real samples, highly selective and stable enzyme biosensors are expected in the future.

## 6. Nano/Microelectrode-Based Catecholamine Monitoring

Monitoring the neurotransmitter molecules released in brain and cell cultures is crucial to building a comprehensive and quick understanding of how, when, and where chemical transmission is taking place. In doing so, microelectrodes have been widely adopted in basic neuroscience and clinical medicine research. When it comes to electrochemical quantification of biomolecules, several nanoelectrodes of various shapes have been developed for in situ measurements. Such nanoscale electrodes were particularly implemented in biology, where multiple biochemical processes require the working electrode to be integrated in vivo, and occasionally in single cells for real-time monitoring [150]. Moreover, an implantable nanoelectrode will ideally serve for the patient’s entire lifetime as in vivo device. Therefore, these miniaturized electrodes at micro-/nanoscale have shown to be favorable for interstitial fluid, and exocytosis events and vesicles monitoring through their implantation in living brain, and inside isolated cells, respectively [151].

The use of carbon fiber microelectrodes (CFME) provides a common means of in vivo detection for a wide range of neurotransmitters, particularly catecholamines, at the molecular level [152]. Deng et al. [153] used the gelatin- and MWCNTs-modified CFME for in vivo monitoring of neurotransmitters with a high level of stability, selectivity, and sensitivity. Furthermore, the use of CFME array for simultaneous and in vivo detection of dopamine and serotonin was also investigated. The suggested method proposes the CFME to be functionalized with diazonium salt, leading to outstanding electrochemical properties, making it possible to quantify the DA and 5-HT levels for different groups of mice [154]. Interestingly, the authors demonstrated that the use of CFME array is more expedient and provides a higher current compared with a single carbon fiber microelectrode.

As an alternative to CFME, the carbon–nanopipette electrode also displays impressive electrochemical properties. By placing a nanopipette electrode slightly above the cellular release site, Hu et al. [155] demonstrated the possibility of quantifying the catecholamines released from individual vesicles as 0.23–1.1 M. Whereas Yang et al. [156] reported the development of cavity carbon-nanopipette electrodes (CNPEs) based biosensor for dopamine quantification in mouse-brain slice. What is more, the nanometers size of CNPEs facilitates their integration into specific narrowed locations for biomolecules quantification such as the level of synapses, and in living cells. Another category of nanoelectrode based on the 3D-printing approach was successfully fabricated using the atomic layer deposition of Al2O3. The fabricated nanoelectrodes have shown an interesting sensitivity toward dopamine stimulation in the adult fly brain via rapid scan cyclic voltammetry. Carbon nanodiamonds was also used for the detection of dopamine released from living cell [157]. It was demonstrated that these nanostructure electrodes are able to be applied for in vivo and in vitro analysis. At the end, the commercialization of microelectrode technology faces many challenges, including problems related to materials, preparation processes, electronic circuit designs, and implantation procedures.

## 7. Advantages and Challenges of Electrochemical Catecholamines Detection

Multiple electrochemical sensing methods based on surface modification with antibodies, enzymes, and nanomaterials have been developed, displaying a rapid, selective and ultralow sensitive detection of biocomponents. Whereas, different techniques was literally performed for porous layer modified sensing electrode, including linear sweep voltammetry, cyclic voltammetry and differential pulse voltammetry, square wave voltammetry taking into account the alteration in kinetics and modification diffusion regime of the sensing electrode [158]. In general, electrochemical biosensors offer a high level of integration, simple instrumental system, and an instantaneous response toward individual and multi-detection with excellent sensitivity and reproducibility [159]. The electrode surface morphology could significantly affect the detection performance, especially when adopting nanostructured surface. Despite their high surface-to-volume ratio, the reproducibility of nanostructured biosensors is impaired due to the uncontrollable material defect.

**Figure 7:**
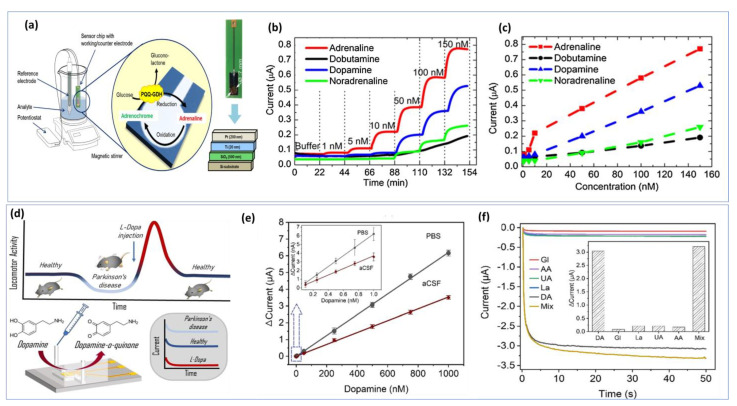
Portable on−chip based NTs biosensor: (**a**) Detection strategy based on bioelectrocatalytical amplification for selective adrenaline detection in human blood plasma. (**b**) Amperometric i−t curves of the biosensor measured in PBS with a variation of catecholamines in the concentration range of 1 nM to 150 nM. (**c**) calibration plots of the biosensor. Reproduced with permission from [162]. Copyright 2023, Elsevier. (**d**) Microfluidic channel based electrochemical detection of dopamine in mouse cerebrospinal fluid and blood. (**e**) Calibration graph performed in PBS or aSCF with an inset showing DA concentration up to 1 nM. (**f**) Selectivity studies of the DA biosensor in the presence of interferants, including glucose, lactate, uric acid, and ascorbic acid. Reproduced with permission from [163]. Copyright 2022, ACS.

Nanoelectrodes-based carbon fibers have contributed significantly to neuroscience by allowing for the detection of electroactive neurotransmitters under very restricted biological conditions [160]. In some cases, several steps are required to synthesize the nanomaterials and nanocomposites to create chemically modified interfaces. To achieve a stable electrode surface, it is essential to maintain the optimal experimental conditions for biosensing events during bio-detection. Any changes in the reaction parameters used in the materials synthesis will be reflected in the electrochemical measurement. However, label-free biosensors offer additional cost-effectiveness because they are easier to develop. In addition to reducing time and steps, label-free detection methods facilitate real-time detection by eliminating expensive labeling protocols [161]. The further development of on-chip electrochemical biosensors is a crucial step toward creating feasible detection strategies suitable for resource-constrained environments. Figure 7a illustrates a simple design for a chip-based epinephrine biosensor. Figure 7b shows the amperometric responses between 1 nM and 150 nM for adrenaline, dobutamine, dopamine, and norepinephrine. The developed approach shows long-term stability and cross-selectivity towards different catecholamines (Figure 7c) [162]. Thus, optimizing the biosensor chip is important for real time monitoring. Additionally, a well-designed microfluid device is necessary for in vitro detection. The miniaturization of the microfluidic chamber and electrode sizes, as well as the decrease in the distance between the three electrodes, allowed the device to achieve a lower detection limit and increased sensitivity (Figure 7d) [163]. Using the chronoamperometry technique with DA concentrations ranging from 0.1 nM to 1 µM prepared in PBS buffer, a linear calibration was achieved. As DA is naturally present in CSF from a mouse model, the electrochemical oxidation of DA in CSF was also investigated (Figure 7e). The current response of the biosensor to DA is linear up to 1000 nM with a detection limit of about 0.1 nM in PBS and CSF. Additionally, good selectivity was demonstrated in Figure 7f.

Some wearable devices are intended to reduce the time between disease diagnosis and effective treatment. This achievement is coupled with the adoption of new technologies like 3D printing of devices microfluidic chips. Taking the example a neurovascular organoid engineering with 3D-printed microfluidic chips was developed by Salmon et al. [164]. More sustained effort is needed to develop flexible electronic materials that can integrate chip technology, IoT, Big Data and artificial intelligence for fully autonomous, self-adaptive, and self-learning AI biosensor systems [165]. In particular, wearable cognitive platforms require flexible neuromorphic devices capable of data processing and arithmetic [166]. Therefore, more technological and research efforts should be directed to the detection of specific low-level neurotransmitters in biofluids and on system integration.

## 8. Other Strategies for Catecholamines Monitoring

### 8.1. Colorimetry and Spectrophotometry

Colorimetric biosensors detect a specific analyte through color changes easily recognized by the naked eye or by simple hand-held optical detectors for quantitative analysis. Various nanostructured materials, including metal nanoparticles [167,168,169,170], metal oxides [171,172,173], carbon and graphene quantum dot [174] metal organic framework [175,176,177] and many others have received much interest as nanozyme-based colorimetric assays for neurotransmitters sensing due to their low cost, intrinsic peroxidase catalytic activity and excellent stability. Recently, the development of multiplexed colorimetric detection platforms has received considerable attention. Accordingly, Jafarinejad et al. [178] proposed an approach for simultaneous colorimetric monitoring of catecholamine neurotransmitters (DA, EP, and NE) ranging from 1 to 30 µg mL^−1^ in urine samples. The detection methodology is based on the formation of silver nanoshells on the surface of gold nanorods using catecholamines as active reducing agents. Experiments have shown that the presence of DA, EP, and NE led to the aggregation of AgNPs on the surfaces of gold nanorods. Three different colors were identified depending on the concentration of neurotransmitters, resulting in a variation of the aspect ratio.

Another approach for a facile and sensitive colorimetric probe was developed by Godoy et al. [179] for the detection of norepinephrine. Benzaldehyde and boronic acid-terminated nanoparticles functionalized with spherical gold nanoparticles are used for the sensing strategy. Synthetic urine was used to test the sensitivity of the probe to NE. In this medium, the limit of detection was 0.09 µM, which is within the range of clinical interest. The development of smart multifunctional bio-nanostructures is a current trend and a future innovative challenge. In this context, a colorimetric smartphone biosensor based on copper oxide nanoparticles detected dopamine with high sensitivity and a low LOD value of 16.9 nM [180]. Therefore, the resulting engineered nanoplatform was determined to be a potential solution for dual-mode colorimetric/electrochemical biosensors.

### 8.2. Surface-Enhanced Raman Spectroscopy (SERS)

Enhanced Surface-enhanced Raman Spectroscopy is one of the most sensitive techniques available for enhancing Raman scattering of molecules through certain nanostructured materials. SERS allows for the structural fingerprinting of analytes at low levels through the plasmonic amplification of electric or chemical fields. Moreover, this technique has been widely applied in potential applications related to surface and biochemistry interface characterization due to its high sensitivity and selectivity. What is more, this technique has been successfully applied to diverse potential applications such as biochemistry, biology, nanotechnology, and especially biosensing based biomedical applications [181]. Due to their distinctive properties, SERS biosensors are attracting a lot of attention. Thus, they can detect molecules with detection limits below nanomolar concentration, high selectivity, excellent sensitivity, and high flexibility. Accordingly, an innovative 2D SERS platform based on the use of Au nanoparticles modified with iron-nitrilotriacetic acid has been developed by Zhou et al. [182] toward the detection of trace levels of epinephrine in the serum. The major achievement of this work was the synthesis of highly stable Au nanoparticles, where PVP prevents the aggregation of Au nanoparticles during the self-assembly process, and the accomplishment of a more uniform distribution of Au nanoparticles at the cyclohexane/water interface. Thus, this approach successfully establishes an interparticle distance, overcoming the difficulty of assembling nanostructures. Through the newly developed SERRS platform, a wide range of EP concentrations was detected in a complex serum medium with high repeatability, sensitivity, and selectivity.

The demonstrated strategy provided promising performance for the detection of targets in various complex domains such as contaminated water, urine, and tissue fluids. Furthermore, Dowek et al. [183] proposed a quantitative and discriminative analysis by surface-enhanced Raman spectroscopy with gold nanoparticles of epinephrine and norepinephrine. This approach allowed for the quantification of EP in the range of concentration from 20 to 80 µg/mL and from 32 to 80 µg/mL for NE.

Additionally, a new liquid-phase SERS detection system was developed using in situ surface modification of Au nanopillar electrodes by Au electrodeposition for the simultaneous detection of DA and UA at 0.1 and 1 nM levels, respectively [184]. Further, Shi et al. [185] used the metal–organic framework (MOF)-loaded silver nanoclusters to develop an aptamer-based DA electrochemical biosensor, which displayed an ultra-low limit of detection of 0.008 nmol L^−1^.

### 8.3. Fluorescence Spectrometry

**Figure 8:**
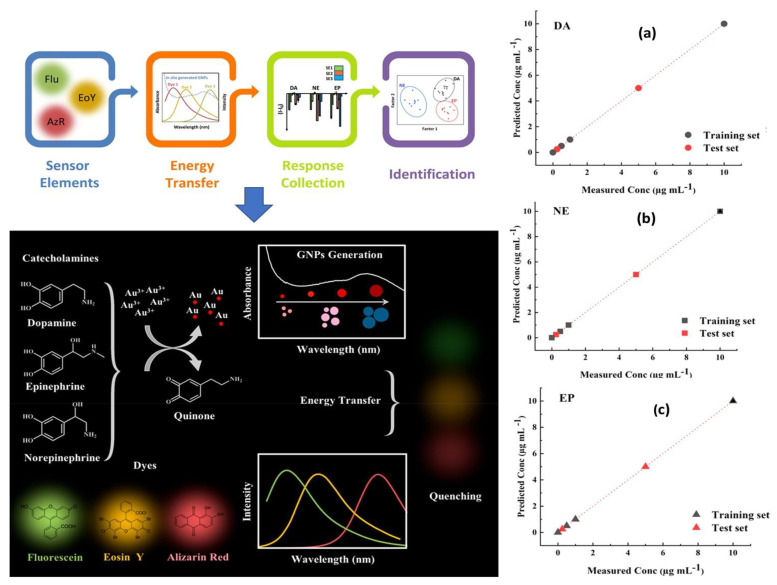
Schematic representation of the working principle and functionalization steps of fluorescence biosensor based AuNPs for simultaneous detection of catecholamine (DA, EP, and NE), and their associated predicted response versus measured concentration of (**a**) Dopamine, (**b**) Norepinephrine, and (**c**) Epinephrine. Reproduced with permission from [188]. Copyright 2020, ACS.

Since fluorescence spectroscopy is more sensitive than UV/Visible, it was recommended for the identification of tiny compounds like neurotransmitters [186]. When it comes to NTs monitoring in real samples and complex biological environment at very low concentrations, using fluorescence spectroscopy has also shown outstanding metrological parameters through minimizing interferences from non-fluorescent compounds. Giving the example of Das and coworkers [187], in which they developed a sensitive and selective fluorescent sensor based on nitrogen doped fluorescent carbon nanoparticles (N–CNPs) for the detection of epinephrine, nor-epinephrine and dopamine. The suggested method was based on the usage of MnO_4_^−^ ion as an analytical signal processor for optimally quenching the fluorescence of N-CNPs and recovering the fluorescence by including the target analytes. The fabricated sensor offers high selectivity and sensitivity toward the above mentioned three catecholamine. Similarly, Fafarinejad et al. [188] also simultaneously detected the DA, EP, and NE using a fluorescent electronic tongue-based AuNPs. Due to the different reducing power of catecholamines, different sized gold nanoparticles were generated with varying levels of aggregation, resulting in different amounts of spectral overlap between the fluorescent dyes and the absorption bands of plasmonic AuNPs generated in situ. In complex biological media, the proposed array performed well at discriminating DA, EP, and NE as shown in Figure 8.

Moreover, An et al. [189] demonstrated a multichannel method for visual monitoring epinephrine, norepinephrine, and levodopa (L-DOPA) based on the in situ synthesis of fluorescent nanoparticles. The detection process was simple and fast, while the reaction of ethylene diamine (EDA) with EP, NE and L-DOPA generates fluorescence of different colors. Additionally, a high selectivity was achieved by testing the influence of interfering substances like serotonin (5-HT), DA, ascorbic acid (AA), and glucose on the sensor’s response. The developed approach proved to be a useful method for introducing an innovative concept for the development of wearable sensors. Gold nanoparticles have proven to be a highly efficient and reliable solution for fluorescence assay in the detection of catecholamines and a variety of biomolecules [190]. The chemical and optical properties of these nanoparticles make them a potential candidate for the highly sensitive and accurate detection of catecholamines at ultra-low levels. Whereas the aggregation of gold nanoparticles presents an additional benefit as it results in changes of geometry, shape, size, and color during the assay which greatly influences the sensing efficiency [191]. Altogether, make the assay process based on Au and Ag nanoparticles more sophisticated, accurate and dependable. Therefore, they are widely used in in vitro and in vivo monitoring at very low levels. Mitra et al. have controlled the size of the synthesized AuNPs through the manipulation of 3-APTMS/3-GPTMS ratio as reducing agents for the detection of DA in human cerebrospinal fluid. In the presence of DA, the positively charged DA molecule with catechol group can be absorbed onto AuNPs to induce aggregation and consequently weaken the absorption band. Low detection limit was found to be 0.63 nM with a linear range from 5 μM to 60 μM [192]. Furthermore, the fluorescence signal was enhanced by the incorporation of gold-silver nanoclusters using protein stabilized agent [193]. Additionally, quantum dots are well-established as labels for fluorescent imaging due to their brightness and resistance to photobleaching. Their biocompatibility, non-toxicity, and water solubility, offer the quantum dot unique opportunities in the field of in vivo optical monitoring. Devi and his coworkers [194] have demonstrated a new methodology for the development of an effective fluorescent probe for non-enzymatic label-free detection of DA using tungsten disulfide.

**Figure 9:**
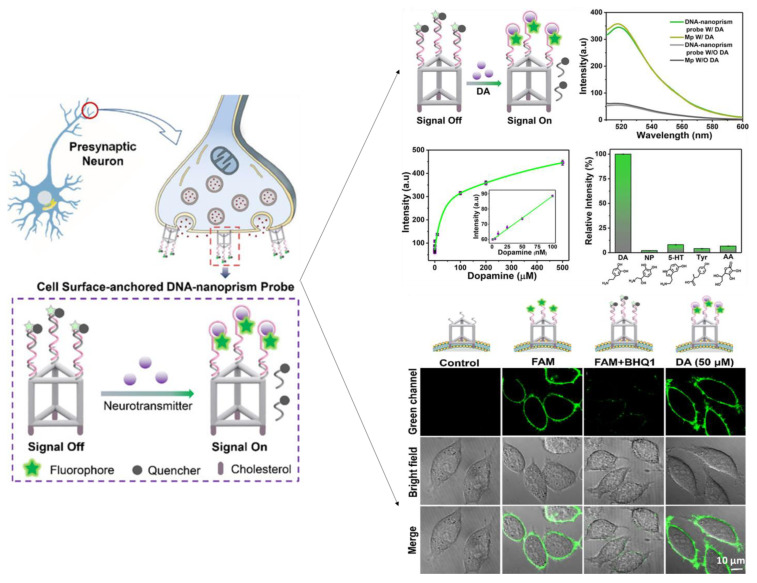
Schematic presentation of fluorescent biosensor with a Cell-Surface-Anchored DNA-Nanoprism for dopamine quantification release from live-cell. Reproduced with permission from [195]. Copyright 2020, ACS.

Zeng et al. [195] developed a DNA-nanoprism fluorescent probe for direct imaging of catecholamine in a single live cell. The well-defined geometric framework enables the self-assembled DNA nanoprism to be highly functionalized with cholesterol labels for the screening of dopamine (Figure 9). Rapid in situ capture of the released DA dissociated the quencher-tagged strand and rapidly produced a fluorescent signal. The DNA nanoprism sensor can be used as a powerful and flexible single-cell imaging nanoplatform of NTs released at the single-cell level due to its outstanding biocompatibility, remarkable cell surface binding durability, and rapid response.

### 8.4. Electrochemiluminescence (ECL) Spectrometry

**Figure 10:**
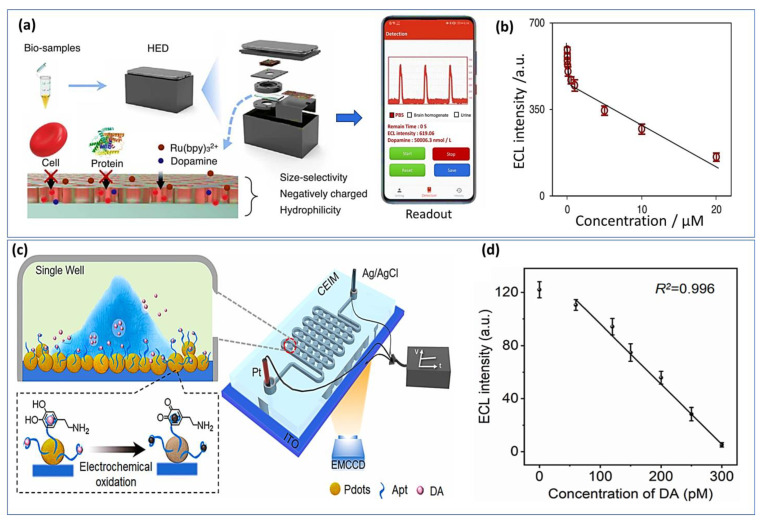
Handheld ELC imaging systems: (**a**) Schematic representation of a handheld ECL analysis device displays the pretreatment and testing of dopamine biosamples, (**b**) Correlation between ECL intensity and DA concentration. Reproduced with permission from [198]. Copyright 2023, Elsevier. (**c**) Experimental setup of confined ECL imaging chip (CEIM) for sensing DA released from a single PC12 cell, (**d**) Calibration curve ECL signal as a function of the DA concentrations. Reproduced with permission from [199]. Copyright 2023, Elsevier.

Electrochemiluminescence (ECL) has attracted significant focus based on its flexibility, simplified setup, and good spatial and temporal control properties. ECL sensors have recently been fabricated using various nanomaterials, which have been introduced due to the rapid advancement in nanoscience and nanotechnology. Using metal organic gels (MOGs) as porous and soft-hybrid supramolecular materials, Wang et al. [196] developed an electrochemiluminescence sensor for epinephrine detection. Since MOGs have unique optical properties, they have been widely studied in a variety of fields. The ECL sensor provides a wide linear range from 10 nM to 1.0 mM and a very low detection limit of about 0.52 nM. Additionally, sulfur quantum dots (SQDs) were used as luminophore for the constraction of ELC sensor for dopamine sensing [197]. Nevertheless, these approaches-based nanomaterials have shown a good sensibility and selectivity, handled and portable ELC sensor remains challenging. For this, recently portable ECL device for POC testing was developed for real time monitoring of dopamine. A miniaturized electrochemical cell for the ECL reaction, an electrical circuit module for voltage stimulation, a silicon photomultiplier module for ECL detection, and a Bluetooth-compatible smartphone for package control comprise the system (Figure 10a). Using this system, the concentration of dopamine was monitored in urine and in vivo rat brains as presented the calibration curve in Figure 10b. An interferant limit of detection was achieved at about 3.5 nM with a range of concentration from 5 nM to 20 μM [198].

Furthermore, a novel ELC imaging chip was constructed for the measurement of dopamine levels in vivo based on aptamer embedded polymer functionalized indium tin oxide electrode surface (Figure 10c) [199]. The handled chip has demonstrated high repeatability with relative standard deviations (RSDs) of below 3.4% and a very low detection limit of about 53 pM (Figure 10d). Other molecules such as AA, tyrosine, and noradrenaline did not affect the sensor response and no significant signal was observed with an excess of those interferents. Li et al. designed for the first time an aptasensor ECL approach for the determination of catecholamine. A DNA-aptamer was used as a molecular recognition element. DA has been sensitively detected in nanomolar range from 1.0 to 50 nM with LOD of 0.32 nM [200]. Another group has proposed a new strategy based on immobilization of the luminophores on the porous ITO electrode as a new feasible solution to overcome the aggregation of perylene diumide derivatives, which opened new perspectives for the development of high-performance ECL sensors [201].

### 8.5. Surface Plasmon Resonance (SPR)

This technique is based on the properties of surface plasmons, which are propagative surface waves located at the interface between a dielectric medium and a metal.

The detection is performed in real-time and without labeling, which is an advantage over other techniques, such as fluorescence [202]. Recently, only a few studies have demonstrated the use of the SPR-based platform for the detection of catecholamines. Among them, Pathak et al. [203] have fabricated a surface plasmon resonance based dopamine biosensor using surface imprinted multiwalled carbon nanotubes and Polypyrrole (PPy/MWCNTs).

Then, to minimize the interference of coexisting molecules like uric acid, ascorbic acid, and epinephrine, a Nafion membrane was dropped over the PPy/MWCNT probe. The developed biosensor shows good sensitivity and selectivity for real time detection in cerebrospinal fluid (CSF) with LOD calculated to be 18.9 pM, which is the lowest reported in the literature. Each of these approaches has drawbacks such as low selectivity, difficult instrumentation, and laborious sample preparation. Nevertheless, remarkable progress has been made, as demonstrated by the increasing number of publications dealing with new or improved detection set-ups. Hence, several commercialized biosensors have been successfully applied in the food industry [204], environmental protection [205] and medicine [206], however most of them are quite cost-intensive. The integration of the biosensor and its associated electronics in a single chip, resulting in a robust, user-friendly, and durable device, is a major research focus in the future [207].

## 9. Conclusions and Future Trends

Nanomaterials have presented a wide range of applications in electrochemical biosensing, as they have become the most promising candidate materials used to modify electrodes. Thanks to their outstanding properties, i.e., their dimensions and morphology, have made them widely used and promoted in research areas such as immobilization of biological molecules, electrochemical reaction catalyzation, electron transfer acceleration, and enhancement of sensor sensitivity. Combining nanomaterials and biotechnology is currently cutting-edge research and a hot topic in the international biotechnology research field. However, it would be beneficial if safer and more biocompatible nanomaterials could be synthesized in the future to be used with biosensors. Until nowadays, several different strategies have been developed for the quantification of catecholamine NTs. However, simpler, more accurate, robust, and inexpensive catecholamine biosensors are still needed. Future studies may focus on creating a fully autonomous portable device, such as an electric chip or paper lab-on-a-chip, that can be operated by patients at their bedside. The term “Lab on a Chip” (LOC) refers to the miniaturization of laboratory instruments into chip-sized devices. With these platforms, chemical processes for electrochemical detection can be miniaturized, integrated, automated, and parallelized. Additionally, miniaturized LOC devices have the advantage of being cost-effective due to the reduced use of reagents in modularly manufactured devices. Another key benefit of LOC devices is that paralleling reaction chambers in a small footprint accelerates the monitoring process. Moreover, LOC technologies provide the ability to further automate analytical systems and increase throughput. Moreover, a continuous research program devoted to microfluidic processes, microfabrication, cost-effective materials, and electronics is necessary in order to stimulate the development of faster, smaller, and less expensive sensing devices. Furthermore, the miniaturization of electrochemical cells and the deployment of autonomous handheld readers are also imperatives for improving POC system efficiency, as well as ensuring full integration, automation, large-scale miniaturization, flexibility, and cost reductions required by large-scale production. Researchers have always focused on developing devices that are user friendly, fast response, and can be operated by non-specialists, using terms such as “point-of-need”, “point-of-care”, “lab-on-chip”, “handled biosensors”, and “microfluidics device”. Nevertheless, producing an optimal device can be time-consuming and expensive. Finally, it is essential to combine biosensors with the Internet of Things (IoT) and communication technologies (ICT) to obtain population health data to predict disease occurrence.

## Data Availability

Not applicable.

## Abbreviations

NTs	Neurotransmitters
CNS	Central nervous system
DA	Dopamine
Ep	Epinephrine
NE	Norepinephrine
5-HT	Serotonin
UA	Uric Acid
AA	Ascorbic acid
GCE	Glassy carbon electrode
CPE	Carbon paste electrode
PGE	Pencil graphite electrode
ITO	Indium Tin Oxide
SWV	Square wave voltammetry
DPV	Differential pulse voltammetry
CV	Cyclic voltammetry
EIS	Electrochemical impedance spectroscopy
LSV	Linear sweep voltammogram.
CuNPs	Copper nanoparticles
RC	Renewable carbon
CNTs	Carbon nanotubes
SWCNTs	Single-Walled Carbon Nanotubes
CQDs	Carbon quantum dots
CuO	Copper oxide
PANi	Polyaniline
GO	Graphene oxide
rGO:	Reduced graphene oxide
PBS	Phosphate-buffered saline
FTO	Fluorine-doped tin oxide
MoS_2_	Molybdenum disulfide
LSGE	Laser-scribed graphene electrode
ZnO	Zinc oxide
LOD	Limit of detection
Ce-MOF	Cerium metal-organic framework
Cu-MOF	Copper metal-organic framework
GCSC	Grass carp skin collagen
FET	Field-effect transistor
TEPA	Tetraethy lenepentamine
CuAlO_2_	Copper aluminate
POC	Point of care
IoT	Internet of Things
AI	Artificial intelligence
3-APTMS	3-aminopropyltrimethoxysilane
3-GPTMS	3-glycidoxypropyltrimethoxysilane
